# Population pharmacokinetics of mycophenolate mofetil in pediatric patients early after liver transplantation

**DOI:** 10.3389/fphar.2022.1002628

**Published:** 2022-10-13

**Authors:** Yinyi Wei, Dongni Wu, Yiyu Chen, Chunqiang Dong, Jianying Qi, Yun Wu, Rongda Cai, Siru Zhou, Chengxin Li, Lulu Niu, Tingqing Wu, Yang Xiao, Taotao Liu

**Affiliations:** ^1^ Department of Pharmacy, The First Affiliated Hospital of Guangxi Medical University, Nanning, China; ^2^ Department of Organ Transplant, The First Affiliated Hospital of Guangxi Medical University, Nanning, China

**Keywords:** mycophenolate mofetil, population pharmacokinetics, liver transplantation, pediatric pharmacology, nonlinear mixed-effect modeling

## Abstract

**Objective:** To investigate the factors influencing the pharmacokinetics of mycophenolate mofetil (MMF) in pediatric patients after liver transplantation, and to establish a population pharmacokinetics model, which can provide a reference for clinical dosage adjustment.

**Methods:** A prospective study in a single center was performed on pediatric patients who were administrated with mycophenolate mofetil dispersible tablets (MMFdt) for at least 4 days after liver transplantation continuously. Blood samples were collected in ethylene diamine tetraacetic acid anticoagulant tubes before dosing and 0.5, 1, 2, 4, 8, and 12 h after the morning intake of MMFdt. The concentrations of mycophenolic acid (MPA) in plasma were assayed with a validated reverse-phase high-performance liquid chromatography method. *UGT1A8 518C > G*, *UGT1A9 -275T > A*, *UGT1A9 -2152C > T*, *UGT2B7 211G > T*, *SLC O 1B1 521T > C* polymorphism were determined by Sanger sequencing. Nonlinear mixed effects modeling was used to establish the population pharmacokinetics (PPK) model. The predictability and stability of the model were internally evaluated by the goodness of fit plots, visual prediction check, normalized prediction errors, and bootstraps.

**Results:** A two-compartment model with first-order absorption and first-order elimination was established with 115 MPA concentrations from 20 pediatric patients. The final model were: CL/F (L/h) = 14.8×(WT/7.5)^0.75^×(DOSE/11.16)^0.452^×е^0.06^, Ka (h^−1^) = 2.02×(WT/7.5)^−0.25^, Vc/F (L) = 6.01×(WT/7.5), Vp/F (L) = 269 (fixed), Q/F (L/h) = 15.4×(WT/7.5)^0.75^×е^1.39^. Where CL/F was the apparent clearance rate, Ka was the absorption rate constant, Vc/F was the apparent distribution volume of the central compartment, Vp/F was the apparent distribution volume of the peripheral compartment, Q/F was the atrioventricular clearance rate, WT was the body weight of the subject, and DOSE was the MMFdt administered dose. The model indicated there was large inter-individual variability in CL/F and Q/F after multiple dosing of MMFdt. Internal evaluation results showed that the final model had good stability and prediction performance.

**Conclusion:** A stable and predictive population pharmacokinetic model of MMFdt in pediatric patients after the early stage of liver transplantation was established. The pediatric patient’s weight and the dose of MMFdt can be a reference to adjust the MMFdt dose.

## Introduction

With the rapid improvement of medical technology, liver transplantation has become an effective therapy for some end-stage liver diseases. Since 2001, Mycophenolate Mofetil (MMF) has been approved by the U.S. Food and Drug Administration for preventing acute rejection after liver transplantation in adults, and the combination of MMF, tacrolimus and glucocorticoid drugs has become the preferred immunosuppressive regimen in most medical centers at present (Perito et al., 2019; Hart et al., 2020). Clinical randomized double-blind trials have confirmed that MMF combined with Calcineurin inhibitors (CNIs) can significantly reduce the incidence of acute rejection of liver and kidney transplantation, while combined with low-dose MMF can reduce the side effects of CNIs and enhance the immunosuppressive effect, thus improving the long-term survival rate after transplantation (Miller et al., 2000; Squifflet et al., 2001; Takada et al., 2013).

As an active metabolite of MMF, Mycophenolic acid (MPA) is affected by plasma albumin (ALB) binding level, gene polymorphism connected to drug metabolism and transport, enterohepatic circulation, body weight, etc., making MPA has complex and variable pharmacokinetics (PK) characteristics *in vivo* (Jing, 2014; Bergan et al., 2021). A growing body of research indicates that MPA PK has significant inter-individual and intra-individual differences. (Shaw et al., 2003; Lobritto et al., 2007; Rong et al., 2021). Under the same dose, there was a tenfold difference in plasma concentrations between individuals, and the difference in area under the concentration-time curve (AUC) can attain fivefold (Staatz and Tett, 2007).

Currently, the recommended effective treatment window for MPA-AUC_0-12_ is 30–60 mg h/L in the liver transplant population. It has been reported that when the MPA-AUC_0-12_ in plasma is lower than 30 mg h/L, it is associated with acute rejection; while when the MPA-AUC_0-12_ is higher than 60 mg h/L, it can increase the incidence of adverse reactions such as diarrhea and myelosuppression (Bergan et al., 2021). Individualized MMF doses may help reduce potential toxic effects and improve clinical outcomes in pediatric liver transplant patients.

Up to now, many studies have explored the population pharmacokinetics (PPK) characteristics of MMF in kidney transplants, liver transplants, heart transplants, and other groups. However, studies based on Asian pediatric liver transplant patients have not been reported. Especially, the use of mycophenolate mofetil dispersible tablet (MMFdt) related PPK research has not been reported. Given the narrow therapeutic window of MPA, it is particularly important for the treatment of the disease and the growth and development of children to get safe and effective plasma concentrations. Therefore, it is urgent to carry out research on PPK in pediatric liver transplant patients.

The purpose of this study was to establish the MMFdt PPK model in pediatric liver transplant patients, which could explore various potential factors on MMFdt PPK, to provide a reference for the individualized medication of MMFdt.

## Methods

### Study design and patients

Pediatric liver transplant patients with deceased or living donor between 2020 and 2021 at the First Affiliated Hospital of Guangxi Medical University were enrolled in this prospective study. The inclusion criteria for this study were: 1) age <18 years; 2) liver transplantation for the first time; 3) MMFdt, tacrolimus, and methylprednisolone as triple immunosuppressive regimen; 4) treatment with MMFdt for more than 4 days. The exclusion criteria were: 1) severe gastrointestinal disease or diarrhea; 2) received combined organ transplantation. Due to the unconfirmed safety and efficacy in the pediatric population and the absence of clear dosing recommendations for pediatric liver transplant patients, clinicians based on clinical experience, postoperatively initially administered the drug through oral or nasal feeding, with an initial dose of 10–15 mg/kg, q12h, and adjusted the dose according to the actual situation. All protocols were approved by the independent Clinical Research Ethics Committee of The First Affiliated Hospital of Guangxi Medical University, and all participants provided written informed consent before enrolment.

The mean half-life (T_1/2_) of MPA was about 17h, so we decided to start sampling after it reaching steady state (4th day). Blood samples were routinely collected on ethylenediamine tetraacetic acid (EDTA) by central venous catheterization before and 0.5, 1, 2, 4, 8, and 12 h after administration. The samples were centrifuged at 3,000 rpm for 3 min immediately after sampling, then the plasma was frozen at -80°C until the analysis.

### MPA assay

Plasma concentrations of MPA were determined by automatic two-dimensional liquid chromatography (2D-HPLC, Demeter Instrument Co. Ltd., Hunan, China). Chromatographic conditions: the first-dimensional column: Aston SC2 (3.5 mm × 25 mm, 5 μm, ANAX, China), mobile phase: 10 mmol/L acetic acid solution-acetonitrile-isopropanol, flow rate: 0.4 ml/min; Intermediate column: Aston SBX4 (3.0 mm × 10 mm, 5 μm, ANAX, China), mobile phase: purified water; The second-dimensional column: Aston SCB (4.6 mm × 125 mm, 5 μm, ANAX, China), mobile phase: methanol, flow rate: 1.2 ml/min. Column temperature: 45°C. The detection wavelength was 304 nm.

Before the determination, 600 μL deproteinizing agent ACP-1B was added into a 1.5 ml EP tube, and then added 200 μL plasma sample exactly. After 1 min of vortex oscillation, high-speed centrifugation was performed for 8 min (14,500 r/min), 650 μL of supernatant was added to 65 μL of ACG-1 protectant into the injection flask, and the sample was shaken and mixed to be measured.

The sample size was 200 μL. The calibration range was 0.3–30.0 mg/L, The lower limit of quantitation (LLOQ) was 0.3 mg/L. The relative standard deviation (RSD) of intra- and inter-day precision is less than 5%. The recovery rate is more than 95%. The stability of the samples stored at room temperature for 24 h and -20°C for 30 days was investigated. The results showed that the stability of MPA in plasma was satisfied and the RSD was less than 5%.

### Processing of data below the quantization limit

For the measured values below the quantization limit (BQL), the M3 method performs best in theory, which maximizes the likelihood for all the data treating BQL observation as censored. However, it can significantly increase the running time and then lead to operation process interruption and estimation failure. Therefore, the M5 method was preferred in this study to process BQL data, that is, to replace BQL with 1/2 of the LLOQ. In addition, double-panel visual predictive check plots were used to evaluate the above data processing methods (Bergstrand and Karlsson, 2009).

### DNA extraction and SNP genotyping

Nucleic acids were extracted with nucleic acid extraction and purification reagent (20190719) from Baiao Technology Co. Ltd. (Shanghai, China) and amplified by ABI 9700-PCR (Applied Biosystems). The assessment of the polymorphisms *UGT1A8 518C > G*, *UGT1A9 -275T > A*, *UGT1A9 -2152C > T*, *UGT2B7 211G > T* and *SLC O 1B1 521T > C* was performed by Sanger sequencing. The primers for the loci mentioned above were synthesized by Tsingke Biotechnology Co., Ltd. (Nanning), which refers to the data provided in the literature (Yu et al., 2017; Wang et al., 2021).

### Population pharmacokinetic analysis

PPK analysis was performed using nonlinear mixed effects modeling NONMEM (version 7.4.3 ICON Development Solution, Ellicott City, MD, USA) approach, facilitated by Perl-speaks-NONMEM (PSN) and Wings for NONMEM (Nick Holford, University of Auckland, New Zealand). The first-order conditional estimation with interaction method (FOCE-I) was selected to estimate the parameters and variability throughout the model-building procedure. ADVAN2 TRANS2 and ADVAN4 TRANS4 were compared in the base model determination.

Model selection criteria were: 1) the value of the objective function (OFV) was minimized; 2) the relative standard error percentage (RES%) of fixed effect parameter and random effect parameter were less than 30% and 50% respectively; 3) the condition number was less than 1,000; 4) the goodness-of-fit (GOF) was improved. Covariates were tested in a univariate fashion and included in the model if the OFV decreased by > 3.84 (*p* < 0.05, χ^2^ distribution, d*f* = 1). After the inclusion of all significant covariates, the significance of the covariates was tested by removing each covariate, and the final model retained the covariates that increased the OFV by > 6.64 (*p* < 0.01, χ^2^ distribution, d*f* = 1) or >9.21 (*p* < 0.01, χ^2^ distribution, d*f* = 2).

### Statistical analysis

Analysis results were expressed as the median and interquartile range (IQR). Hardy Weinberg equilibrium was applied to assess the deviation of allele and genotype frequencies. All statistical analyses were performed with IBM SPSS Statistics Version 22.0 (SPSS Inc., Chicago, IL, United States).

### Model validation

Model internal validation was conducted by GOF plots, visual prediction check (VPC), normalized prediction distribution errors (NPDE), and bootstrap to evaluate the stability and prediction performance.

## Results

### Patient demographics

Altogether 20 pediatric liver transplant patients with 122 samples were included. The main demographic characteristics of the study population were listed in Table 1, as results shown in the patient’s biochemistry were within the normal range except for liver function.

**TABLE 1:** Demographic characteristics and Laboratory test results of pediatric patients.

Characteristics	Median (Q1-Q3)	Range
Sex: male/female (n/n)	12/8	—
Age at inclusion (years)	0.74 (0.61–1.69)	0.42–7.76
**Age at inclusion, n (%)**		
<24 months	15	—
2–10 years	5	—
Height (cm)	67.5 (62.2–80.0)	58.0–119.0
Weight (kg)	7.5 (6.0–10.0)	4.6–27.0
BSA (m^2^)	0.39 (0.32–0.43)	0.28–0.94
Liver donor: living/deceased (n/n)	11/9	—
**Indication for liver transplantation, n (%)**		
Liver cirrhosis after Kasai operation	16	—
Biliary atresia	1	—
Liver failure	1	—
Hepatoblastoma	1	—
Glycogen storage disease	1	—
GRWR (%)	3.6 (2.5–4.6)	1.0–6.0
POD (days)	12 (10–14)	4–39
Dose (mg/kg^/^dose)	11.2 (10.0–15.0)	8.9–61.5
PLT (10^9^/L)	203.8 (131.6–320.4)	42.0–671.9
TBiL (μmol/L)	13.65 (7.40–22.45)	2.60–593.50
DBiL (μmol/L)	8.70 (5.35–16.0)	0.40–73.80
TP (g/L)	50.40 (45.84–55.40)	3.10–65.50
ALB (g/L)	35.20 (32.45–37.25)	24.30–47.50
AST (U/L)	41.0 (26.5–66.0)	11.0–1,523.0
ALT (U/L)	65.0 (27.0–133.0)	2.0–2,286.0
CCR (ml/min)	106.80 (84.85–135.40)	44.90–360.90
Combination drugs, n (%)	—	—
Meropenem	11 (55)	—
Voriconazole	10 (50)	—
Fluconazole	4 (20)	—
Linezolid	5 (25)	—
Lansoprazole	6 (30)	—
Furosemide	9 (45)	—

BSA, body surface area, BSA (m^2^), $\sqrt[{2}]{\frac{Height\left(cm\right)\times Weight\left(kg\right)}{3600}}$ ; GRWR, graft to recipient weight ratio; POD, post operative days; PLT, platelet; TBiL, total bilirubin; DBiL, direct bilirubin; TP, total protein; ALB, albumin; AST, aspartate aminotransferase; ALT, alanine aminotransferase; CCR, creatinine clearance.

**FIGURE 1:**
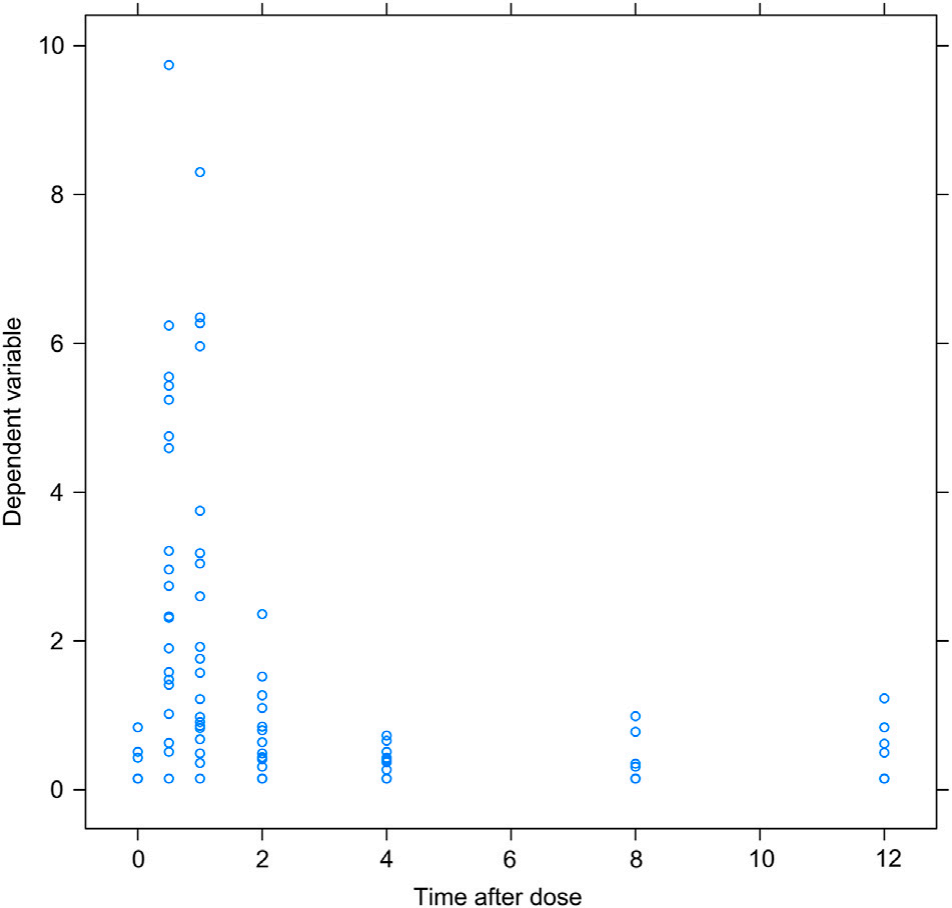
Scatter plot of MPA Observations vs. Time after last dose.

Of the 122 samples, 7 concentration values below the detection limit were directly removed as missing data. The scatter plots of the remaining 115 concentration values and the time after the last dose were shown in Figure 1, from which it could be seen that the first peak of MPA blood concentrations were mainly concentrated in 0.5–2 h, and no obvious secondary peak of MPA blood concentrations caused by enterohepatic circulation (EHC) was observed. Among 115 concentration values, 21 concentration values were lower than LLOQ, accounting for 18.3%. Hence, the M5 method was used in this study to replace all 21 BQL data with 0.15 mg/L.

**FIGURE 2:**
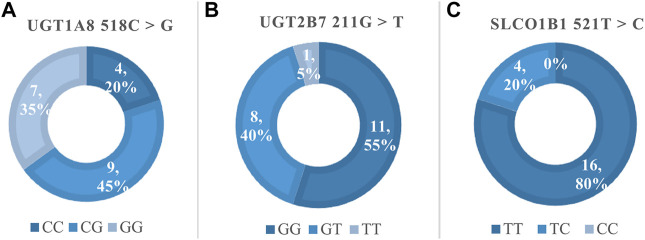
The genotype frequencies of MPA related polymorphisms. **(A)** Frequency of UGT1A8 518C > G, Hardy-Weinberg *p* > 0.05 (χ^2^ = 0.13); **(B)** Frequency of UGT2B7 211G > T, Hardy-Weinberg *p* > 0.05 (χ^2^ = 0.09); **(C)** Frequency of SLCO1B1 521T > C, Hardy-Weinberg *p* > 0.05 (χ^2^ = 0.25).

Figure 2 showed the frequency distribution of specific genotypes in the 20 pediatric liver transplant patients. Genotype and allele frequencies were not significantly different than expected, which represented the population was in Hardy-Weinberg equilibrium.

### Population pharmacokinetic model

At last, a two-compartment pharmacokinetic model with first-order absorption was selected to describe the data. In the random effects model, both the inter-individual variation (IIV) and residual variation (RV) were represented as exponents for the best-fitting effect.

The OFV value of the model including the allometric scaling model decreased by 10.061. The dose of MMFdt was retained in the model as a covariate significantly affecting CL/F. The screening process of the covariate model were shown in Table 2, and the final model formula were as follows:

CL/F (L/h)=14.8×(WT/7.5)∧0.75×((DOSE/11.16)∧0.452)×е∧0.06 (1)

Ka (h∧(−1))=2.02×(WT/7.5)∧(−0.25) (2)

Vc/F (L)=6.01×(WT/7.5) (3)

Vp/F (L)=269 (fixed) (4)

Q/F (L/h)=15.4×(WT/7.5)∧0.75×е∧1.39 (5)

**TABLE 2:** he process of the final model.

No.	Description	OFV	∆ OFV	*p* value
Base model		-74.659	—	—
Model 1	Add allometric scaling on base model	-84.724	10.065	<0.01
Stepwise forward 1				
Model 2	Add DOSE on CL in Model 1	-91.465	6.741	<0.01
Model 3	Add ALT on CL in Model 1	-89.543	4.819	<0.05
Model 4	Add UGT1A8 on Q in Model 1	-90.894	6.170	<0.05
Model 5	Add SLCO1B1 on Q in Model 1	-89.092	4.368	<0.05
Model 6	Add GRWR on CL in Model 1	-89.013	4.289	<0.05
Stepwise forward 2				
Model 7	Add UGT1A8 on Q in Model 2	-97.246	5.781	>0.05
Model 8	Add SLCO1B1 on Q in Model 2	-95.533	4.068	<0.05
Model 9	Add GRWR on CL in Model 2	-97.825	6.360	<0.05
Stepwise forward 3				
Model 10	Add GRWR on CL in Model 8	-101.862	6.329	<0.05
Backward elimination 1				
Model 11	Remove DOSE from CL in Model 10	-93.440	8.422	<0.01
Model 12	Remove GRWR from CL in Model 10	-95.533	6.329	>0.05
Model 13	Remove SLCO1B1 from Q in Model 10	-97.825	4.037	>0.05
Backward elimination 2				
Model 14	Remove GRWR from CL in Model 11	-89.092	4.348	>0.05
Model 15	Remove SLCO1B1 from Q in Model 11	-89.013	4.427	>0.05

Final parameter estimation, interindividual variability with standard error estimation, and bootstrap statistics of the final PPK model were represented in Table 3.

**TABLE 3:** PK parameter estimates and bootstrap results of final model.

Parameter	Final model			Bootstrap		
	Estimate	RSE (%)	Shrinkage (%)	Median	2.5, 97.5 percentiles	Shrinkage (%)
OFV	-91.5	—	—	-98.5	-162.5, -45.2	—
CL/F (L/h)	14.8	8.5	—	14.5	11.0, 17.6	—
K_a_ (h^−1^)	2.0	18.2	—	2.1	1.4, 3.5	—
V_c_/F (L)	6.0	25.5	—	6.6	3.1, 12.7	—
V_p_/F (L)	269	—	—	269	—	—
Q/F (L/h)	15.4	27.7	—	15.2	6.8, 31.8	—
θDOSE	0.452	20.2	—	0.463	0.183, 0.934	—
IIV CL/F (%)	24.5	26.9	19.8	22.8	8.3, 33.5	22.2
IIV Q/F (%)	117.9	22.1	9.8	115.2	46.4, 161.2	10.6
RV (%)	50.3	7.3	8.2	49.2	41.4, 56.1	7.7

RES, percent relative standard error; θDOSE, typical population value of DOSE; IIV, interindividual variability; RV, residual variability.

### Model evaluation

**FIGURE 3:**
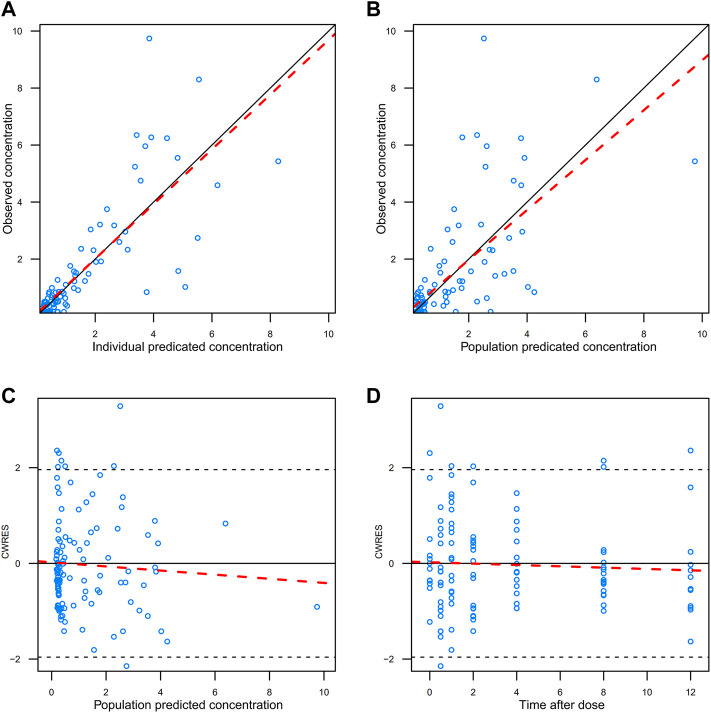
GOFs diagnosis diagram of the final model. **(A)** observed value vs. individual predicted value; **(B)** observed value vs. predicted population value; **(C)** conditional weight residuals vs. population predicted values; **(D)** conditional weighted residuals vs. time after dose. The red dotted line represents trendline, while the solid black line represents reference line.

The GOF plots of the final model was shown in Figure 3. The trend line of individual prediction (IPRED), population prediction (PRED), and dependent variable (DV) of the final model was close to the reference line, which meant the deviation between the predicted value and the measured value was small, and the prediction performance of the model was improved.

**FIGURE 4:**
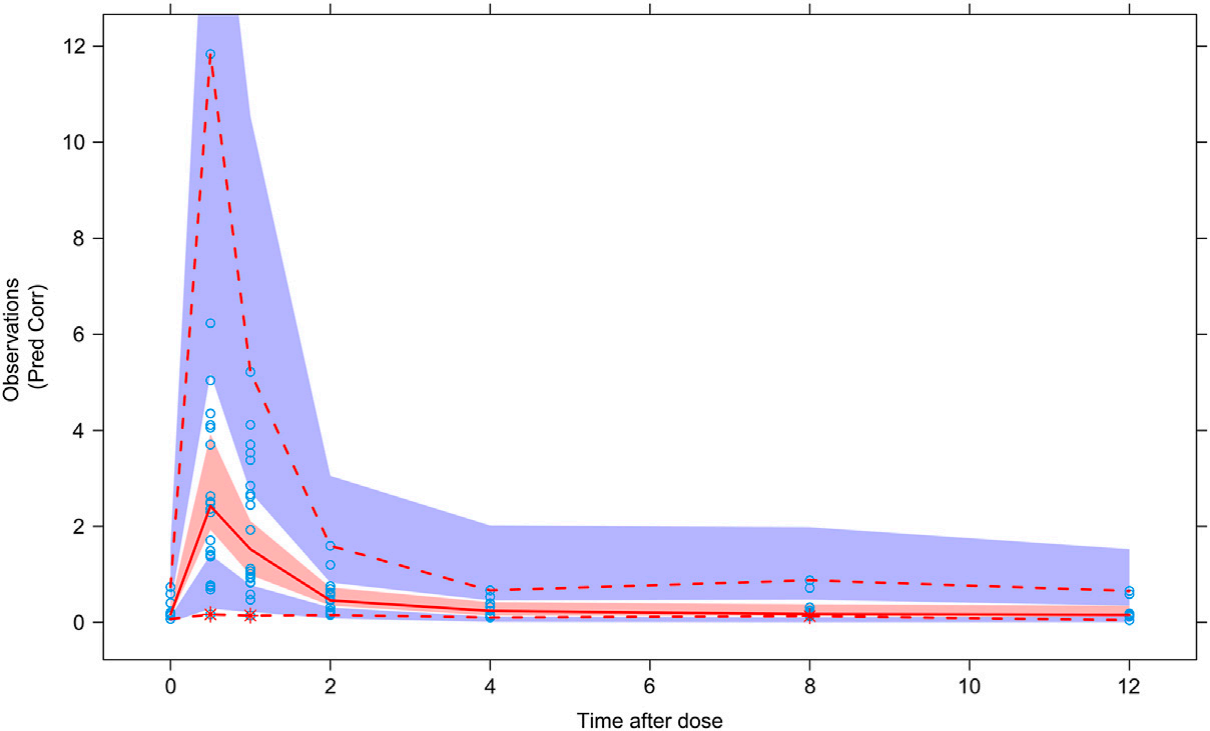
Visual predictive check of the final model. Circles represent observations. Red solid line stands for the median of simulated concentrations, and red imaginary lines stand for the 90% prediction interval (PI) (5%, 95%) of the predictive MPA concentrations. Shaded areas represent the 95% confidence interval (CI) for each line.

The results of VPC verification of the final model were shown in Figure 4, which represented that the model adequately described the overall trend and variability in the observed data. As shown in the figure, the majority of MPA concentration values were within the 90% prediction interval, indicating that the model’s predictive performance was acceptable.

**FIGURE 5:**
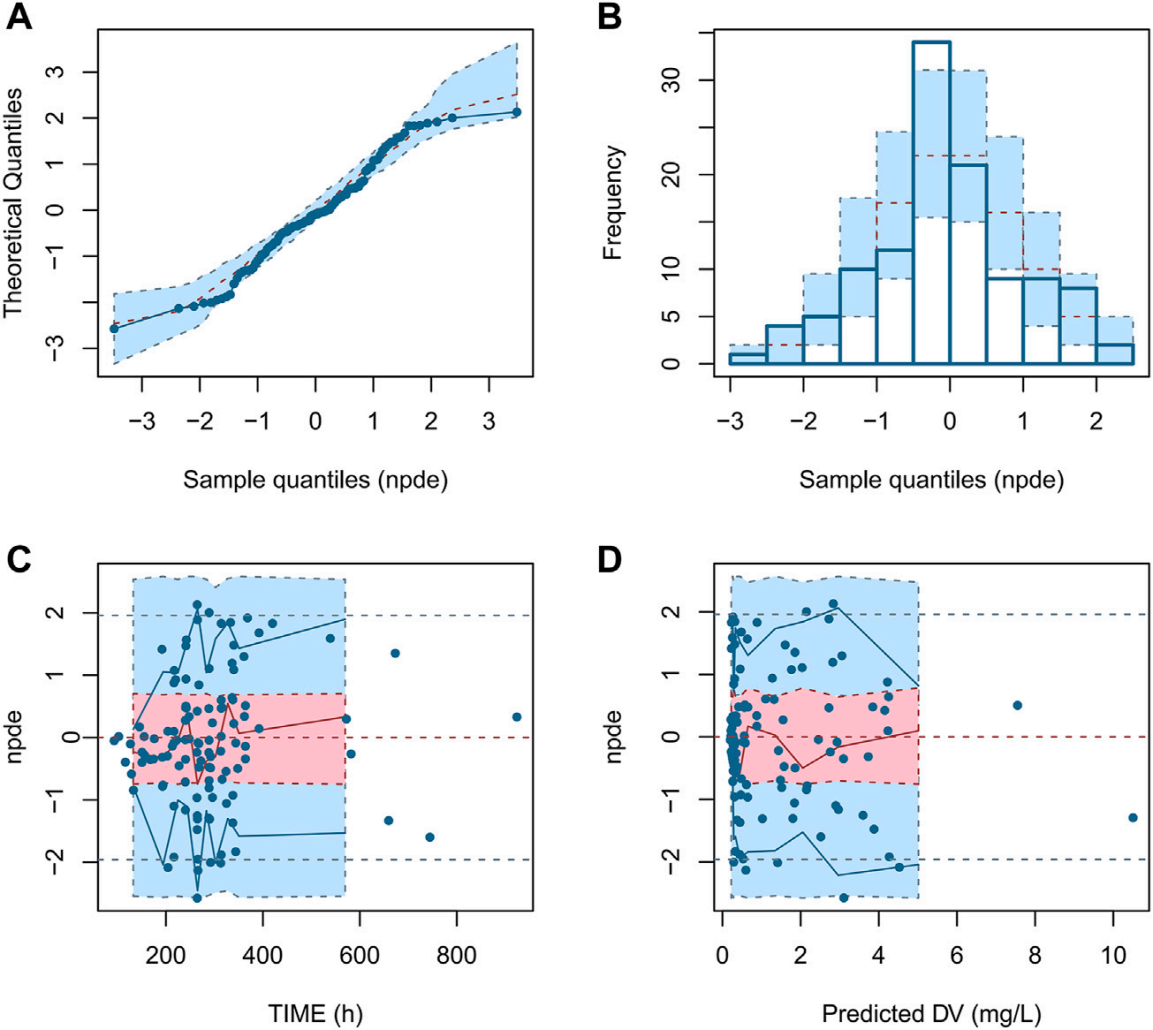
NPDE diagnostic diagram of the final model. **(A)** Q - Q plot of NPDE vs. normalized distribution; **(B)** Distribution diagram of predictive distribution error; **(C)** NPDE vs. TIME; **(D)** NPDE vs. PRED.

The NPDE diagnostic diagram indicates that the final model had an approximately normal distribution trend (Figure 5). The predicted concentrations of NPDE were randomly distributed near the reference line, and most of them were in the acceptable range (±2), and the Global test *p*-value was 0.422 (>0.05), which indicated that the prediction performance of the model was satisfied.

The robustness rate of the final model was 98.1%. The detailed results of bootstrap were set out in Table 3, which indicated the model was reliable with good accuracy and stability.

**FIGURE 6:**
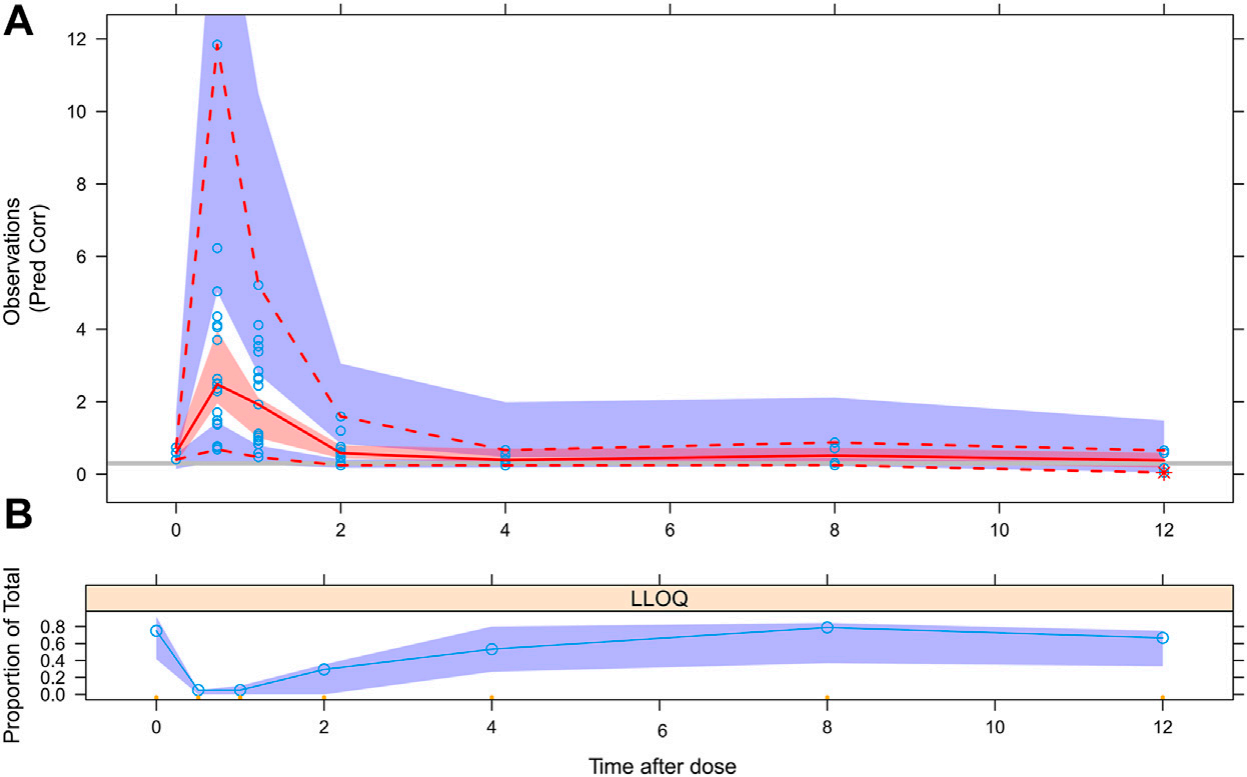
VPC evaluation of BQL data with M5 method. **(A)** Circles represent observations. Red solid line stands for the median of simulated concentrations, and red imaginary lines stand for the 90% PI (5%, 95%) of the predictive MPA concentrations. Shaded areas represent the 95% CI for each line. The horizontal gray line represents the LLOQ. **(B)** Circles represent BQL observations, shaded areas represent the 95% CI for predictive BQL concentrations.

The results of BQL data processing with the M5 method was showed in Figure 6, we divided the blood drug concentration data into two parts: data higher than LLOQ and data lower than LLOQ. It can be seen that the measured BQL data were all located inside the shadow, which showed that the M5 method was suitable for processing BQL data.

## Discussion

In this study, we prospectively gathered data and sought to determine the influence of PPK in pediatric liver transplant patients, establishing a two-compartment model of first-order absorption with body weight and dose as covariates by the NONMEM method. The internal evaluation indicated that the final model had satisfactory predictability, which could provide a reference for MMFdt clinical application.

To describe the *in vivo* process of MPA as accurately as possible, previous researchers mostly used the two-compartment model with a complex absorption or distribution process of MPA in different pediatric groups, such as renal transplantation, hematopoietic stem cell transplantation, and idiopathic nephrotic syndrome. The range of population typical values of CL/F, Ka, Vc/F, Vp/F, and Q/F were12.7–25.3 L/h, 0.39–5.21 h^−1^, 35–411 L, 4.75–64.7 L, and 3.74–113 L/h (Zhao et al., 2010; Sherwin et al., 2012; Dong et al., 2014) respectively. In our study, the typical values of CL/F, Ka, Vc/F, Vp/F, and Q/F in the final model are 14.8 L/h、2.02 h^−1^、6.01 L、269 L, and 15.4 L/h, respectively, which were within the range of population typical values of previous research models. Furthermore, prior studies had noted that body weight, age, and combined use of cyclosporine or tacrolimus were mostly included as covariables in CL/F and Vc/F, among which Q/F showed great variability in estimation (BOV% 26.6%–207.83%), and no covariables could be found to explain the variation (Rong et al., 2021), which was consistent with the results observed in our study. Vp was fixed to 269 L, as it gave the optimal parameter estimation, and the IIV and RV decreased significantly. According to the Vp value of transplant patients reported in previous literature in the range of 137 L–496 L (Rong et al., 2021), the result of our study was reasonable.

In 2012, Barau et al. studied MMF PK for the first time in pediatric liver transplant patients and established the MMF PK model by traditional pharmacodynamic method with the estimated values of CL/F, Vc/F, and Ka being 12.7 L/h、64.7 L and 3.9 h^−1^, respectively. This study indicated that Vc/F decreased with the increase of time after transplantation. The authors believed that the free fraction of MPA (*f*-MPA) increased due to the lower ALB and increased bilirubin level after liver transplantation, increasing Vc/F (Barau et al., 2012). Nevertheless, no significant effect of time after transplantation on MMF PK parameters was found in other pediatric populations. In comparison, the estimated value of Vc/F was significantly different from the results of our study, which may be caused by the weight difference between the two populations. It had been reported that Vc increased exponentially with increasing body weight in adult solid-organ transplant patients (Funaki, 1999), and the median body weight in our study was 7.5 kg while the median body weight reported by Barau et al. was 23.8 kg, which may explain the difference of Vc/F estimates between their and our study.

When shrinkage is higher than 20–30%, diagnostics based on Bayes estimates (EBEs) lack informativeness and may be misleading (Savic and Karlsson, 2009). In our study, the shrinkage values of interindividual variation in CL/F, Q/F, and RV of the final model were 19.8%, 9.8%, and 8.2%, respectively, which were all less than 20%. The diagnostics were relatively reliable in model building and evaluation.

We tried to introduce body weight into the PPK modeling process through the allometric scaling model, and the results showed that the allometric scaling model could significantly improve the fitting effect of the model. This theory-based allometric scaling uses body weight as a power model with an exponent of 0.75 for functional PK parameters (e.g., clearance), and it is considered a proper biologically scaling method to account for different body sizes (Holford et al., 2013).

According to previous studies, the bioavailability of MMF was not constant, and it significantly decreased with the increase in MMF dose, showing nonlinear PK characteristics (Dong et al., 2014; Catic-Dordevic et al., 2021). This may explain the phenomenon that CL/F of MPA increased with the increase of dose in this study. With the dose increased, the absorption of MPA in the intestine reached saturation, while the unabsorbed MPA was directly excreted. Another possible explanation for this was that when the amount of mycophenolic acid glucoside acid (MPAG) reached saturation in the enterohepatic circulation, the use of high-dose MMF would cause more MPAG to be directly excreted through the kidney, and the generation of MPA through the enterohepatic circulation would reduce, leading to MPA exposure reduction (de Winter et al., 2011). It had been reported that due to better absorption of MPA at lower pH, the combined use of proton pump inhibitors (PPIs) could also result in a decrease in the bioavailability of MPA (Bergan et al., 2021). Miura et al. found that MPA AUC was lower when MMF was combined with 30 mg lansoprazole compared to 10 mg rabeprazole or no PPIs (Miura et al., 2008). In addition to interacting with MPA absorption, PPIs can also lead to reductions in MPA, peak concentration, and AUC by inhibiting *ABCB1* mediated transport (Pauli-Magnus et al., 2001; Wedemeyer and Blume, 2014). As regards the effect of dose on bioavailability, it could also be explained by the fact that clinicians may administer a higher dose in consideration of the possibility of higher clearance in some pediatric patients. Overall, data on the bioavailability of MPA are scarce in current studies, and no covariate has been found to explain the observed variation. It is worthy of note that modeling with further consideration of physiological factors may be needed to confirm this hypothesis in pediatric populations. Therefore, the possible impact of dose on the bioavailability of MPA should be considered in clinical therapeutic drug monitoring.

Some studies had shown that the decrease of ALB level in the body and the reduction of its binding to MPA led to the increase of *f*-MPA concentration, which generated an increase in MPA clearance (Weber et al., 2008; Yoshimura et al., 2018). Reviewing the published pediatric population studies, only one study based on the cohort of children with idiopathic nephrotic syndrome included ALB in the final model (Zhao et al., 2010). However, no effect of ALB on MPA clearance was observed in our study, which might be interpreted as the normal level of ALB and renal function in the current study patients, failing to reach the threshold that can cause a significant reduction in MPA exposure (Kim et al., 2012).

Although polymorphisms of *UGT1A8*, *UGT1A9*, *UGT2B7*, and *SLC O 1B1* genes were determined, which were considered to be important effect on the metabolism and transport process of MPA (Kuypers et al., 2005; Jiang and Hu, 2021), none had been verified in pediatric liver transplant patients. Unfortunately, no significant effect of these genotypes on MPA PK were observed in our study. At present, the effect of gene polymorphism on MPA PK is rarely observed in children, which may be because these liver drug enzyme activity of children is significantly lower than that of adults. According to published studies, the abundance of *UGTs* such as *UGT1A9* and *UGT2B7* reaches 50% of adult levels between 2.6 and 10.3 years of age, while the children in our study were all younger than 10 years old, and 75% of them (*n* = 15) were younger than 2 years old. In summary, no significant gene polymorphisms that affected MPA PK were observed in this study, perhaps due to the small sample size, short study duration (<30 d), or the young age of the study population with low metabolic and transporter activity (Miura et al., 2007; Badee et al., 2019).

There were several limitations for the current study. This study was a single-center study with a limited number of participants and only performed model internal evaluation, which should gather further data to expand the sample for a more rigorous external evaluation.

Yet, it is difficult to carry out population pharmacokinetic studies in pediatric liver transplant patients. Thus, the conclusions of this study still require to be further verification. We will continue to gather more patient data to improve the model and explore the PPK characteristics of MPA in pediatric liver transplant patients in China.

## Conclusion

In summary, our study established a population pharmacokinetic model of MMFdt in pediatric patients after early liver transplantation. Based on this PPK analysis, we identified body weight and MMFdt dose as significant covariates for MPA clearance. The model’s internal evaluation methods showed the final model had good stability, reliability, and predictive performance, which might provide a reference for the individualized medication of MMFdt.

## Data Availability

The original contributions presented in the study are included in the article/Supplementary Material, further inquiries can be directed to the corresponding authors.
